# Correction to “Prevalence of Impaired Fasting Glucose (IFG) and Associated Prediabetes Metabolic Markers Among Adolescents in Saudi Arabia”

**DOI:** 10.1155/pedi/9823295

**Published:** 2026-06-14

H. Alfaraidi, A. Babiker, F. Al Juraibah et al., “Prevalence of Impaired Fasting Glucose (IFG) and Associated Prediabetes Metabolic Markers Among Adolescents in Saudi Arabia,” *Pediatric Diabetes* 2026 (2026): 5568644, https://doi.org/10.1155/pedi/5568644.

In the article titled “Prevalence of Impaired Fasting Glucose (IFG) and Associated Prediabetes Metabolic Markers Among Adolescents in Saudi Arabia,” there was an error in Affiliation 5. The correct affiliation is shown below:

Division of Pediatric Endocrinology, King Abdullah Specialized Hospital, National Guard Health Affairs, Al Qassim, Saudi Arabia

We apologize for this error.

